# Supplementary material for: Leukemic stem cells activate lineage inappropriate signalling pathways to promote their growth
Source: Nat Commun. 2024 Feb 14;15:1359. doi: 10.1038/s41467-024-45691-4 (PMC10867020; doi:10.1038/s41467-024-45691-4)
Supplement: Supplementary file 1 — Supplementary Information [file 41467_2024_45691_MOESM1_ESM.pdf]

Supplementary Information to:

Leukemic stem cells activate lineage inappropriate signalling pathways to promote their growth

Supplementary Figure 1: Subtype specific gene expression and chromatin accessibility is established in LSCs

Supplementary Figure 2: t(8;21) AML LSCs are differentially signalling responsive

Supplementary Figure 3: Aberrant VEGF and IL-5 signalling in t(8;21) AML drives LSC activation

Supplementary Figure 4: VEGFA and IL5RA inhibitors reduce patient-derived xenograft proliferation

Supplementary Figure 5: VEGF and IL-5 signals terminate at the AP-1 family of transcription factors

Supplementary Figure 6: AP-1 orchestrates a shift in transcriptional regulation from an LSC to a blast pattern

Supplementary Figure 7: AP-1 is required for maintenance of the blast program

Supplementary Figure 8: The signalling response of t(8;21) cells operates within a RUNX1::ETO dependent regulatory circuit and ectopic activation of signalling pathways is also found in other AML subtypes

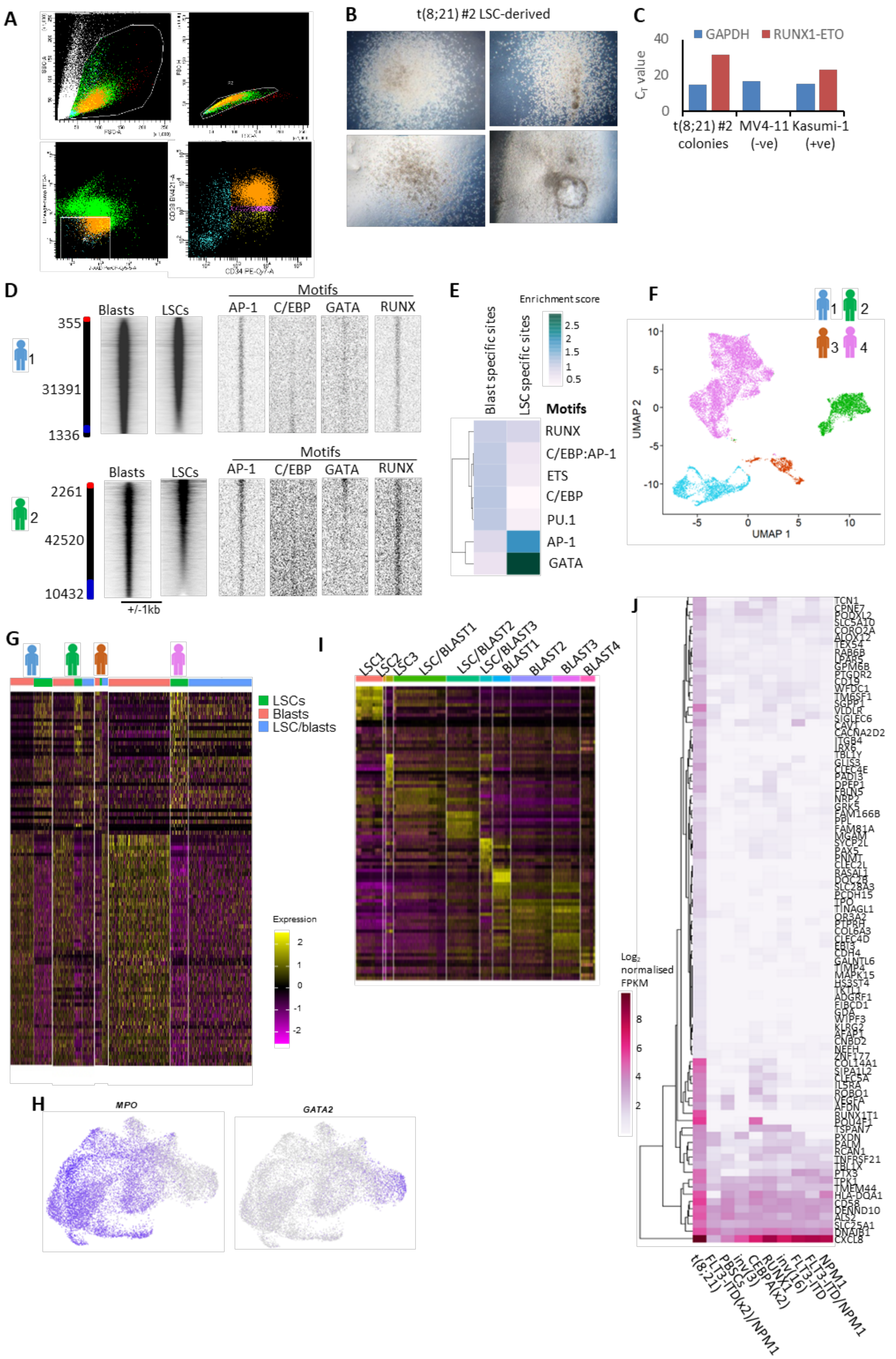
A
B
t(8;21) #2 LSC-derived
C
GAPDH
RUNX1-ETO
CT value
40
20
0
t(8;21) #2 colonies
MV4-11 (-ve)
Kasumi-1 (+ve)
D
Blasts
LSCs
Motifs
AP-1
C/EBP
GATA
RUNX
355
31391
1336
1
2261
42520
10432
2
+/-1kb
E
Enrichment score
2.5
2
1.5
1
0.5
Blast specific sites
LSC specific sites
Motifs
RUNX
C/EBP:AP-1
ETS
C/EBP
PU.1
AP-1
GATA
F
1
2
3
4
UMAP 2
UMAP 1
10
5
0
-5
-10
G
LSCs
Blasts
LSC/blasts
Expression
2
1
0
-1
-2
H
MPO
GATA2
I
LSC1
LSC2
LSC3
LSC/BLAST1
LSC/BLAST2
LSC/BLAST3
BLAST1
BLAST2
BLAST3
BLAST4
J
Log2 normalised FPKM
8
6
4
2
TCN1
CXCL8
t(8;21)
FLT3-ITD(x2)/NPM1
PBSCs
inv(3)
CEBPA(x2)
RUNX1
inv(16)
FLT3-ITD
FLT3-ITD/NPM1
NPM1

Supplementary Figure 1: Subtype specific gene expression and chromatin accessibility is established in LSCs

(A) Dot plots showing the gating strategy for cell sorting in Figure 1, cells were captured by forward/side scatter area (top left), doublets removed with forward scatter area/height (top right), then dead and lineage positive cells removed with lineage cocktail-FITC/7-AAD (bottom left) and finally CD34+CD38+ (orange) and CD34+CD38- (yellow) cells sorted (bottom right). **(B)** Light microscope images of four colonies formed from t(8;21) #2 LSCs, no colonies were formed from the blasts nor from any other patient. **(C)** qRT-PCR was performed on the colonies in B, RUNX1::ETO was detected with primers which span the breakpoint albeit at a lower expression level than Kasumi-1 cell line, whilst no transcript was detected in MV4-11 as a negative control. N=1 biological replicates, bars shown the mean of 2 technical replicates **(D)** ATAC-seq on sorted LSCs and blasts was ranked by fold change of the tag count at distal peaks and represented as density plots (+/1kb of the summit). The blue bar indicates blast specific sites and the red bar LSC specific sites where the normalised tag-count of specific sites is at least two-fold different. Transcription factor binding motif frequencies are plotted along the same axis across the same window. **(E)** A motif enrichment score was calculated based on motif frequency in the LSC and blast specific sites calculated from the merged ATAC-seq data from t(8;21) #1 and #2. **(F)** UMAP plot of merged scRNA-seq, with contributing cells coloured according to the patient of origin **(G)** Heatmap showing the genes identified as comprising a t(8;21) LSC and blast specific signature in LSCs and blasts of each patient. **(H)** Expression of *GATA2 and MPO* projected onto the UMAP plot, where blue indicates the normalised UMI count. **(I)** Heatmap showing the top 10 most upregulated marker genes identified in each scRNA-seq cluster. **(J)** Heatmap showing the log_2 normalised FPKM of t(8;21) specific genes in AML with different driver mutations and healthy CD34+ PBSCs[7].

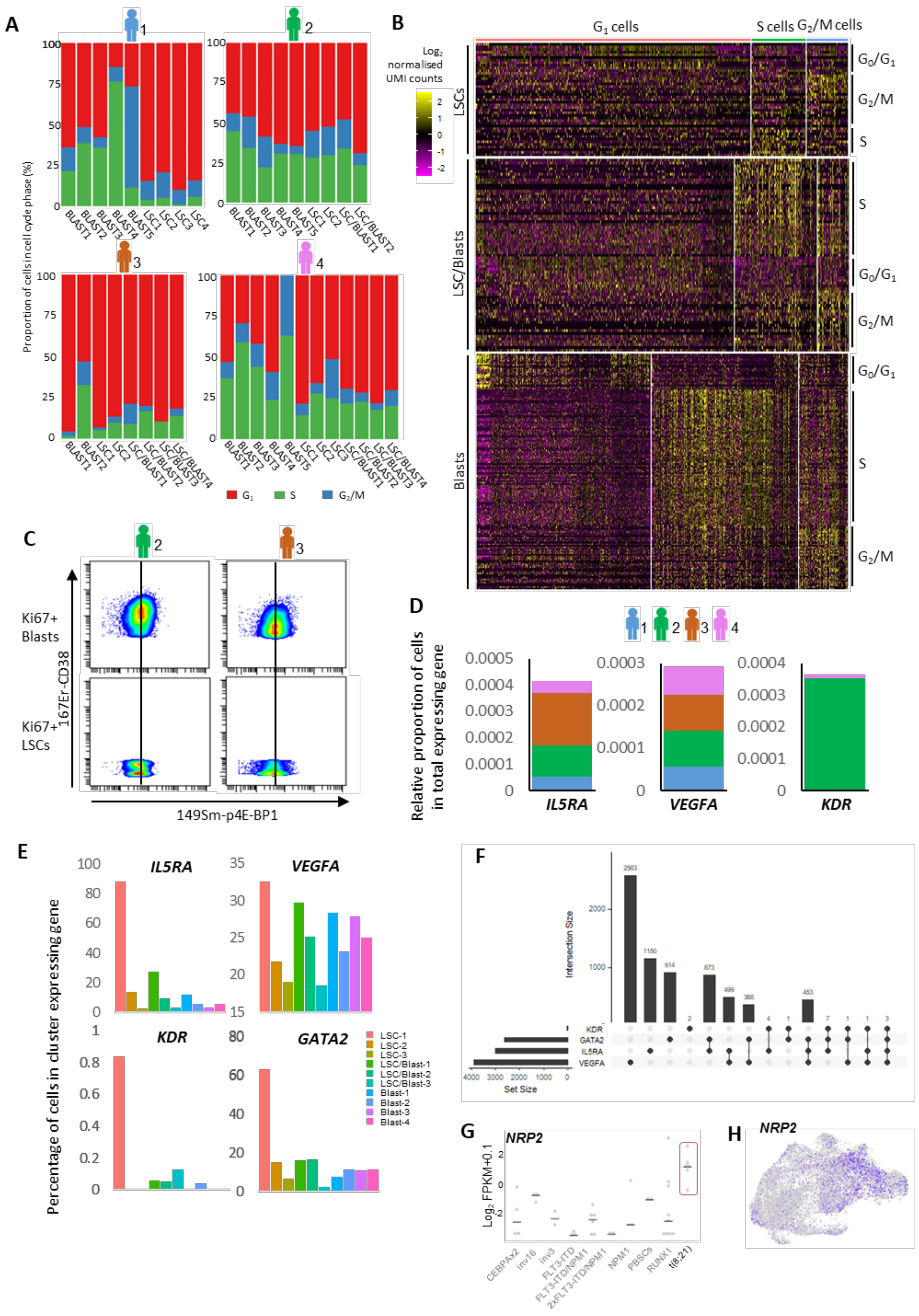

Supplementary Figure 2: t(8;21) AML LSCs are differentially signalling responsive

(A) The proportion of cells in each cluster assigned to each cell cycle stage by their gene expression pattern. **(B)** Heatmaps showing the expression in scRNA-seq of marker genes for each cell cycle phase, plotted according to the cells assigned to each cell cycle phase in LSCs, blasts and LSC/blast transition cells. **(C)** Biplots showing mass cytometry data for CD38 vs p4-EBP1 in Ki67+ CD34+/CD38+ blasts (top) and Ki67+ CD34+/CD38- LSCs (bottom), the vertical line indicates the median in the blasts. **(D)** Relative proportion of cells expressing *VEGFA, IL5RA* and *KDR,* shown as number of gene X expressing cells per patient/total number of cells per patient/total number of gene X expressing cells. **(E)** Bar charts indicating to which population the cells expressing the genes belong. **(F)** Upset plot showing the number of cells co-expressing *VEGFA, IL5RA, GATA2* and *KDR*. **(G)** Normalised $\log_2$ FPKM of *NRP2* in AML with different driver mutations and healthy CD34+ PBSCs[7]. Horizontal bars indicate the median of all samples. N = 4 CEBPAx2, 3 inv(16), 2 inv(3), 3 FLT3-ITD, 2 FLT3-ITD2x/NPM1, 3 NPM1, 2 PBSCs, 9 RUNX1 and 5 t(8;21) patients. **(H)** Expression of *NRP2* projected onto the UMAP plots, where blue indicates the normalised UMI count.

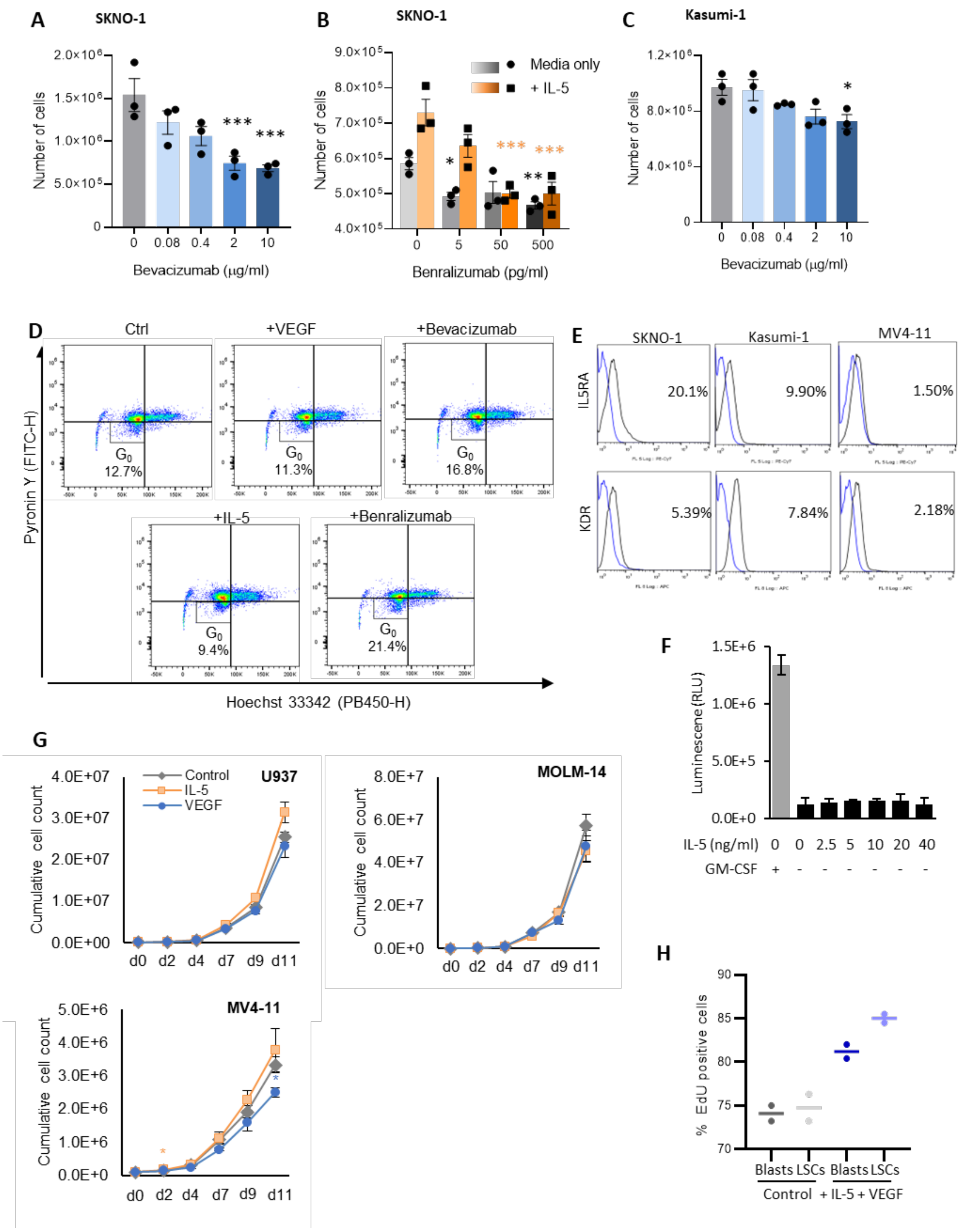

Supplementary Figure 3: Aberrant VEGF and IL-5 signalling in t(8;21) AML drives LSC activation

(A & C) Dose response showing the number of SKNO-1 **(A)** and Kasumi-1 **(C)** cells after 6 days of treatment with four doses of bevacizumab or with no bevacizumab. **(B)** Dose response showing the number of SKNO-1 cells after 6 days of treatment with benralizumab, +/- IL-5. **(A-C)** n=3 wells. **(D)** Representative dot plots from three independent experiments showing the proportion of cells in G_0

(Hoechst low, Pyronin Y negative) in SKNO-1 following 24 hours of treatment with VEGF, IL-5, bevacizumab or benralizumab **(E)** Cell surface expression of IL5RA and KDR was measured by flow cytometry in SKNO-1, Kasumi-1 and MV4-11 cells, 2 hours after serum stimulation, representative histograms with isotype control from 4 independent experiments are shown. **(F)** Dose response showing the luminescence (in relative luminescence units) as determined by celltiter glo at 6 concentrations of IL-5 in the absence of GM-CSF, n=3 wells. **(G)** Growth curves were performed by growing U937, MOLM-14 or MV4-11 cells for 11 days, counting and passaging every 2-3 days. Cells were grown in the presence of IL-5 or VEGF-165. Each point represents the mean of 3 wells, and error bars show SEM. **(H)** Percentage EdU positivity of PKH-26-LSCs/Claret-Blasts or PKH-26-Blasts/Claret-LSCs, re-combined and cultured for 6 days with or without IL-5 and VEGF-165. Horizontal bar indicates mean. **(A-C & F)** * indicates $p < 0.05$, ** $p < 0.01$, *** $p < 0.005$ using Student's T-tests vs controls. Source data are provided as a Source Data file.

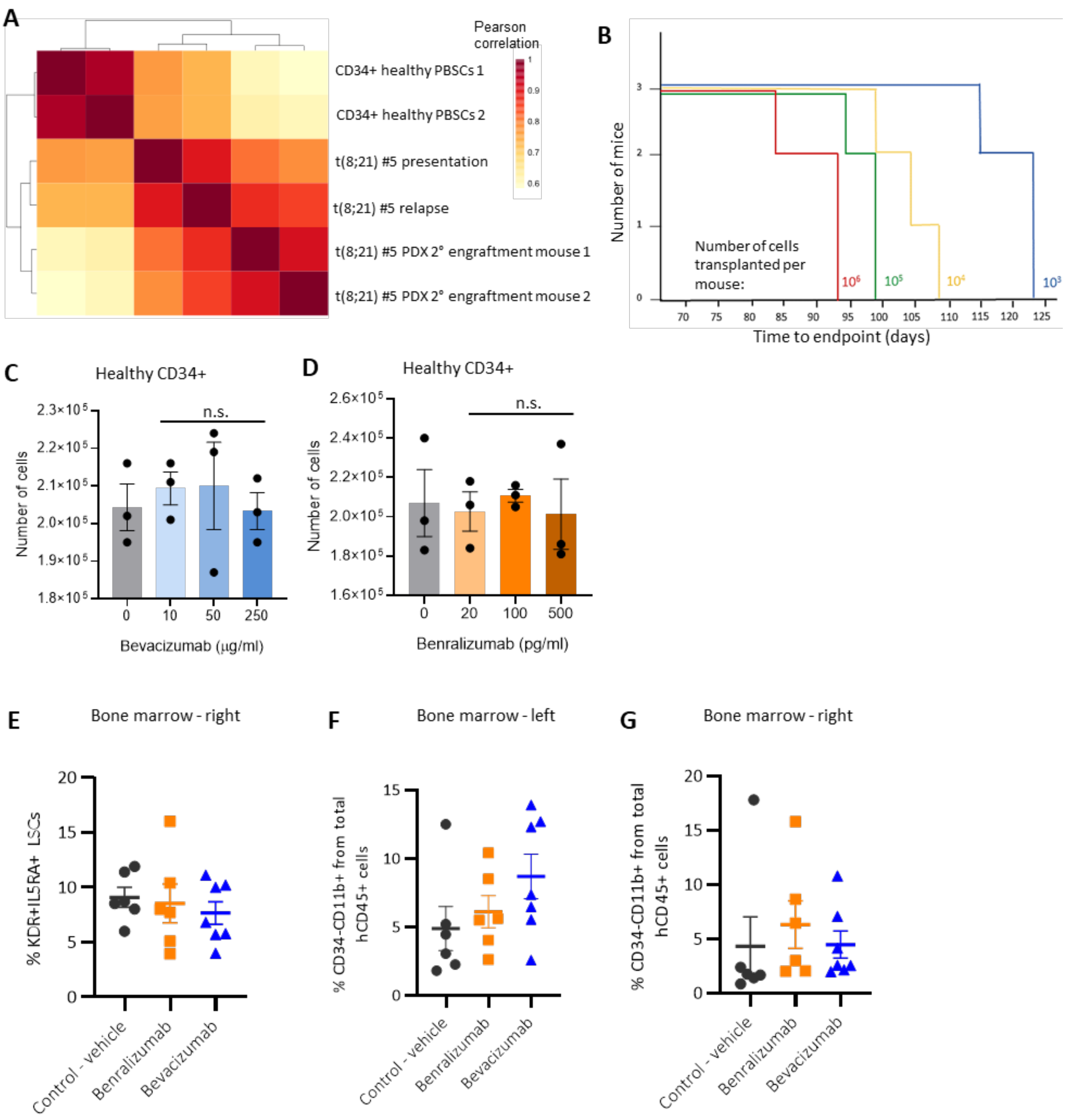

Supplementary Figure 4: VEGFA and IL5RA inhibitors reduce patient-derived xenograft proliferation

(A) Heatmap with hierarchical clustering showing the Pearson correlation values between gene expression data from RNA-seq on two healthy CD34+ PBSC samples, CD34+ sorted patient cells from t(8;21) #5 at presentation and on relapse, and from two mice which were secondarily engrafted with t(8;21) #5 PDX (**B**) Three mice were engrafted with primary PDX cells at 10^3, 10^4, 10^5 and 10^6 cells per mouse and the survival time until humane end-point recorded **(C-D)** Dose response showing the number of healthy CD34+ cells after 6 days of treatment *in vitro* with 3 doses of bevacizumab **(C)** or with 3 doses of benralizumab (+10ng/ml IL-5) **(D),** bar height shows the mean of 3 wells and error

bars indicate SEM. **(E)** Percentage of KDR and IL5RA positive hCD45+/CD34+/CD38- LSCs in right bone marrow at end point. **(F-G)** Percentage of hCD45+/CD34-/CD11b positive cells in left **(F)** and right **(G)** bone marrow at end point. **(E-G)** Horizontal and error bars show mean and SEM of the mice in each treatment group as Figure 1. Source data are provided as a Source Data file.

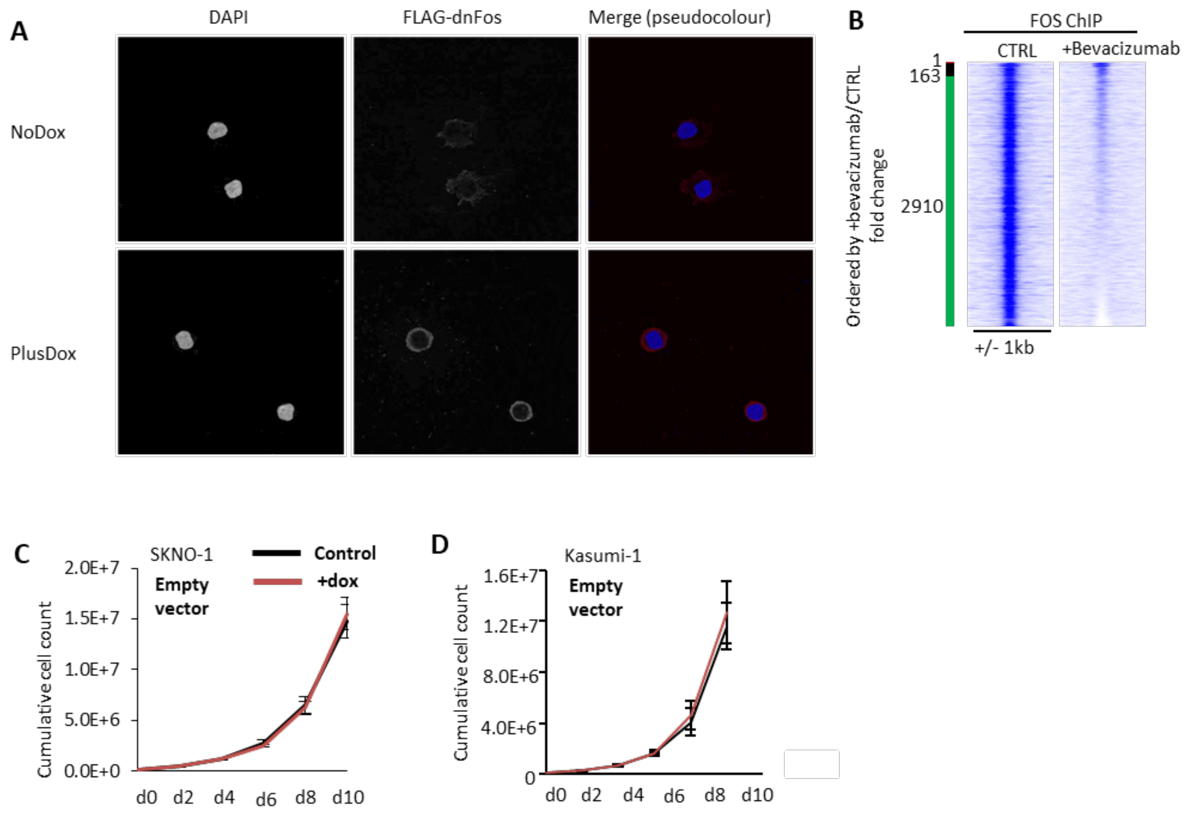

Supplementary Figure 5: VEGF and IL-5 signals terminate at the AP-1 family of transcription factors **(A)** Immunofluorescence images of dnFOS induction in Kasumi-1 cells by staining for the flag-tag, counterstained for DAPI. Separated colour channels are shown in grey scale whilst the pseudocolour is both colour channels combined. **(B)** Density plot showing FOS ChIP signal (+/-1kb from the peak summits) at all sites ranked by the fold change of the tag counts between treated with bevacizumab and an untreated control. The green bar indicates sites which were at least 2-fold reduced with bevacizumab and the red bar indicates the site which was at least 2-fold higher with bevacizumab. **(C-D)** Growth curves were performed by growing SKNO-1 **(C)** or Kasumi-1 **(D)** cells for 10 days, counting and passaging every 2 days following transduction and selection with an EV control of the dnFOS plasmid, where GFP only is induced by doxycycline. Each point indicates the mean of 3 wells, error bars show SEM, no significant differences were seen at any time point.

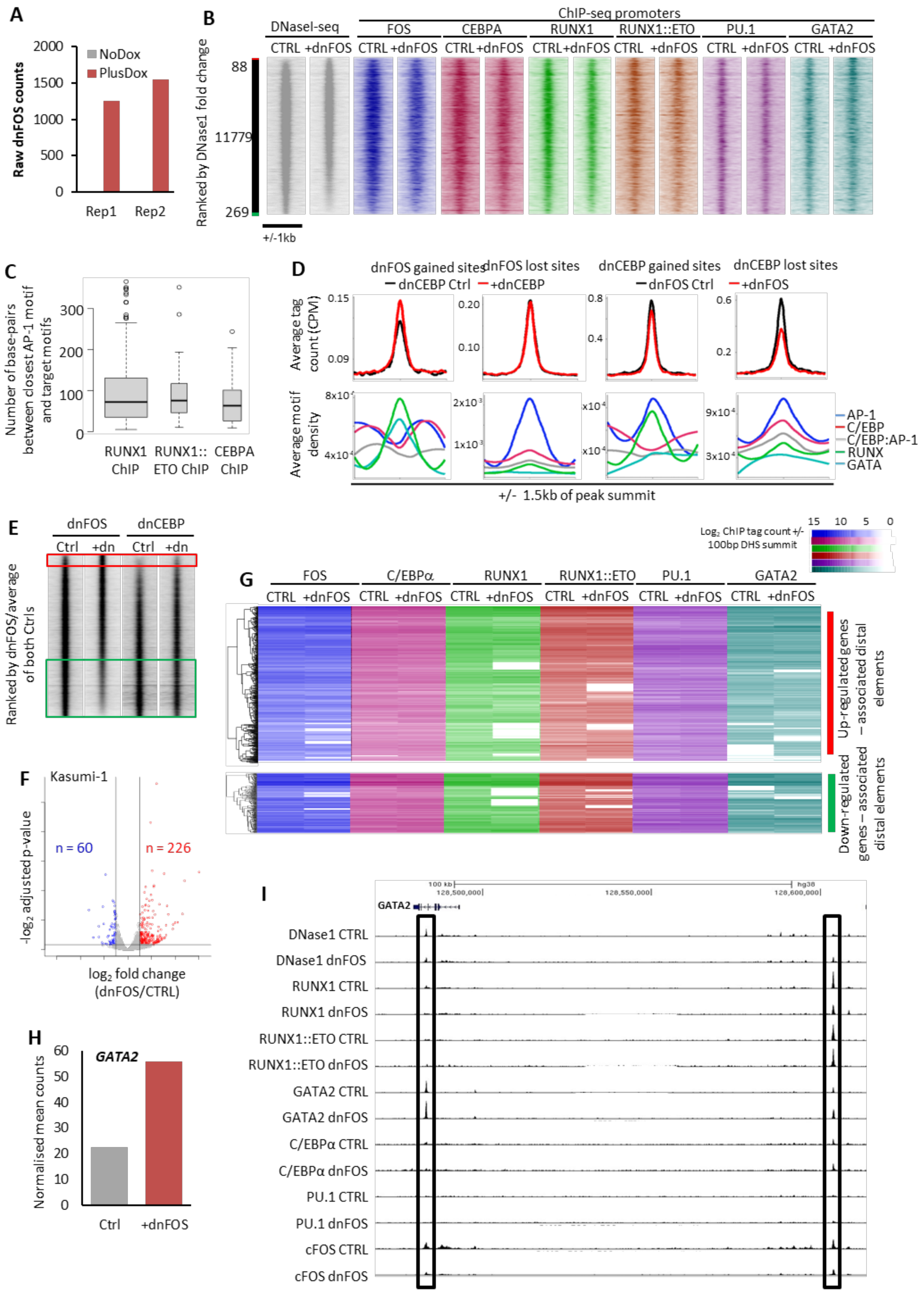

Supplementary Figure 6: AP-1 orchestrates a shift in transcriptional regulation from an LSC to a blast pattern

(A) Bar chart showing the raw dnFOS counts in the two RNA-seq independent experiments (rep1 and rep2). (**B**) DNase1 with and without dnFOS induced by doxycycline in the Kasumi-1 cell line, was ranked by fold change of the tag count at promoters and represented as density plots (+/1kb of the summit). The red bar indicates dnFOS specific sites and the green bar control specific sites where the normalised tag-count of specific sites is at least two-fold different. ChIP data from FOS, CEBPA, RUNX1, RUNX1::ETO, PU.1 and GATA2 with and without dnFOS were plotted on the same axis across the same window. **(C)** The proximity of AP-1 motifs to RUNX motifs in RUNX and RUNX1::ETO ChIP control specific sites, or to CEBP motifs in CEBPA ChIP control specific sites was calculated and presented as boxplots where the bold line indicates the mean and the error bars indicate 95% confidence intervals. GSEA analysis was used to compare blast and LSC specific genes which were at least 2-fold differentially expressed in bulk RNA-seq with the ranked fold change gene expression from Kasumi-1 with induction of dominant negative CEBP. **(D)** Average profiles showing the normalised tag count of dnFOS DNaseI at dnCEBP 2-fold specific sites or dnCEBP DNaseI at dnFOS 2-fold specific sites where black shows the controls and red the induced (top) and the average motif density with loess regression at these same sites (bottom). **(E)** Density plots were generated from DNaseI tag count at distal sites, ranked by the fold change of +dnFOS/the average of the dnFOS and dnCEBP controls, +/-1kb of the peak summit, +dnCEBP was plotted on the same axis. **(F)** Volcano plot showing gene expression changes in Kasumi-1 in response to dnFOS induction, vertical lines indicated at least 2-fold change whilst the horizontal line indicates $-\log_2$ adjusted p-value of 0.1, genes which are significantly up-regulated are coloured red, and genes which are significantly down-regulated are in blue. **(G)** The tag count of the ChIPs shown in A was measured at all DHS associated with two-fold differentially expressed genes following dnFOS induction (+/- 100bp of the DHS summit). The tag counts and gene expression fold change are shown as heatmaps, with the DHS and their associated gene expression change subject to hierarchical clustering. **(H)** Bar chart showing the normalised mean counts for *GATA2* expression in Kasumi-1 with and without dnFOS. **(I)** UCSC genome browser screenshot showing DNase1 and ChIP-seq at the *GATA2* locus.

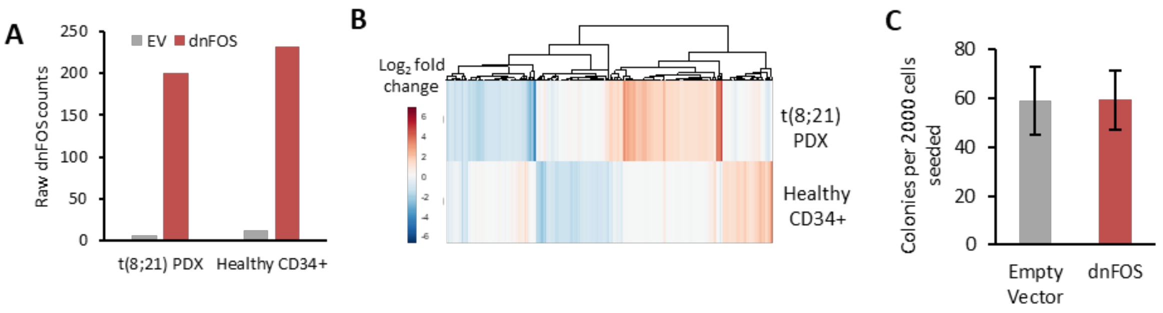

Supplementary Figure 7: AP-1 is required for maintenance of the blast program

(A) Bar graphs showing the raw number of dnFOS counts in RNA-seq in PDX and healthy CD34+ cells with or without induction of dnFOS. **(B)** Heatmap showing log_2 fold changes dnFOS vs EV in the PDX and healthy CD34+ cells for all genes which were at least 2 fold de-regulated in either. **(C)** Colony forming assay in healthy CD34+ PBSCs induced and sorted for dnFOS or EV, n=3 independent experiments.

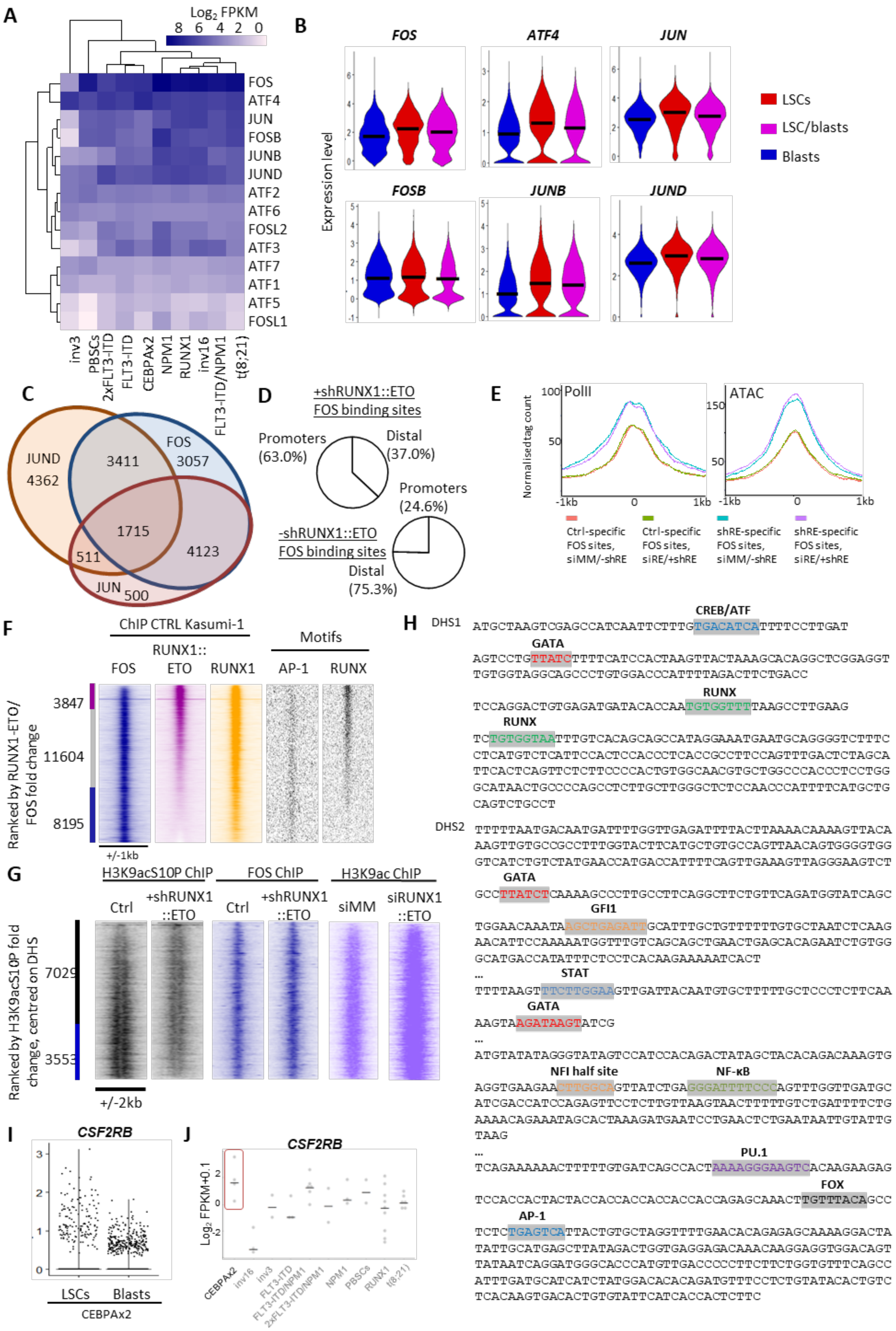
A
Log2 FPKM
8 6 4 2 0
FOS
ATF4
JUN
FOSB
JUNB
JUND
ATF2
ATF6
FOSL2
ATF3
ATF7
ATF1
ATF5
FOSL1
inv3
PBSCs
2xFLT3-ITD
FLT3-ITD
CEBPAx2
NPM1
RUNX1
inv16
FLT3-ITD/NPM1
t(8;21)
B
FOS
ATF4
JUN
FOSB
JUNB
JUND
Expression level
LSCs
LSC/blasts
Blasts
C
JUND 4362
FOS 3057
3411
1715
4123
511
JUN 500
D
+shRUNX1::ETO FOS binding sites
Promoters (63.0%)
Distal (37.0%)
-shRUNX1::ETO FOS binding sites
Promoters (24.6%)
Distal (75.3%)
E
Normalised tag count
PolII
ATAC
-1kb
0
1kb
Ctrl-specific FOS sites, siMM/-shRE
Ctrl-specific FOS sites, siRE/+shRE
shRE-specific FOS sites, siMM/-shRE
shRE-specific FOS sites, siRE/+shRE
F
ChIP CTRL Kasumi-1
Motifs
FOS
RUNX1::ETO
RUNX1
AP-1
RUNX
Ranked by RUNX1-ETO/FOS fold change
3847
11604
8195
+/-1kb
G
H3K9acS10P ChIP
FOS ChIP
H3K9ac ChIP
Ctrl
+shRUNX1::ETO
Ctrl
+shRUNX1::ETO
siMM
siRUNX1::ETO
Ranked by H3K9acS10P fold change, centred on DHS
7029
3553
+/-2kb
I
CSF2RB
LSCs
Blasts
CEBPAx2
J
CSF2RB
Log2 FPKM+0.1
CEBPAx2
inv16
inv3
FLT3-ITD
FLT3-ITD/NPM1
2xFLT3-ITD/NPM1
NPM1
PBSCs
RUNX1
t(8;21)
H
DHS1
CREB/ATF
ATGCTAAGTCGAGCCATCAATTCTTTGTGACATCATTTTCCTTGAT
GATA
AGTCCTGTTATCTTTTCATCCACTAAGTTACTAAAGCACAGGCTCGGAGGT
TGTGGTAGGCAGCCCTGTGGACCCATTTTAGACTTCTGACC
RUNX
TCCAGGACTGTGAGATGATACACCAATGTGGTTTTAAGCCTTGAAG
RUNX
TCTGTGGTAATTTGTCACAGCAGCCATAGGAAATGAATGCAGGGGTCTTTC
CTCATGTCTCATTCCACTCCACCCTCACCGCCTTCCAGTTTGACTCTAGCA
TTCACTCAGTTCTCTTCCCCACTGTGGCAACGTGCTGGCCCACCCTCCTGG
GCATAACTGCCCCAGCCTCTTGCTTGGGCTCTCCAACCCATTTTCATGCTG
CAGTCTGCCT
DHS2
TTTTTAATGACAATGATTTTGGTTGAGATTTTACTTAAAACAAAAGTTACA
AAGTTGTGCCGCCTTTGGTACTTCATGCTGTGCCAGTTAACAGTGGGGTGG
GTCATCTGTCTATGAACCATGACCATTTTCAGTTGAAAGTTAGGGAAGTCT
GATA
GCCTTATCTCAAAAGCCCTTGCCTTCAGGCTTCTGTTCAGATGGTATCAGC
GFI1
TGGAACAAATAAGCTGAGATTGCATTTGCTGTTTTTTGTGCTAATCTCAAG
AACATTCCAAAAATGGTTTGTCAGCAGCTGAACTGAGCACAGAATCTGTGG
GCATGACCATATTTCTCCTCACAAGAAAAATCACT
...
STAT
TTTTAAGTTTCTTGGAAGTTGATTACAATGTGCTTTTTGCTCCCTCTTCAA
GATA
AAGTAAGATAAGTATCG
...
ATGTATATAGGGTATAGTCCATCCACAGACTATAGCTACACAGACAAAGTG
NFI half site
NF-κB
AGGTGAAGAACTTGGCAGTTATCTGAGGGATTTTCCCAGTTTGGTTGATGC
ATCGACCATCCAGAGTTCCTCTTGTTAAGTAACTTTTTGTCTGATTTTCTG
AAAACAGAAATAGCACTAAAGATGAATCCTGAACTCTGAATAATTGTATTG
TAAG
...
PU.1
TCAGAAAAAACTTTTTGTGATCAGCCACTAAAAGGGAAGTCACAAGAAGAG
FOX
TCCACCACTACTACCACCACCACCACCACCAGAGCAAACTTGTTTACAGCC
AP-1
TCTCTGAGTCATTACTGTGCTAGGTTTTGAACACAGAGAGCAAAAGGACTA
TATTGCATGAGCTTATAGACTGGTGAGGAGACAAACAAGGAGGTGGACAGT
TATAATCAGGATGGGCACCCATGTTGACCCCCTTCTTCTGGTGTTTCAGCC
ATTTGATGCATCATCTATGGACACACAGATGTTTCCTCTGTATACACTGTC
TCACAAGTGACACTGTGTATTCATCACCACTCTTC

Supplementary Figure 8: The signalling response of t(8;21) cells operates within a RUNX1::ETO dependent regulatory circuit and ectopic activation of signalling pathways is also found in other AML subtypes

(A) Heatmap with hierarchical clustering showing the average log_2 FPKM of FOS, JUN and ATF transcription factors in 9 subtypes of AML and healthy PBSCs[7]. **(B)** Violin plots showing the expression per cell of the six AP-1 family members most highly expressed in t(8;21) AML, grouped by LSC, blasts and LSC/blast transition cells. The horizontal black bar indicates the median per cell population. All LSC/Blast comparisons had a p-value < $1e^{-50}$ except for *FOSB*. **(C)** Venn diagram showing the overlap in ChIP peaks from FOS, JUN and JUND ChIP-seq in Kasumi-1. **(D)** Pie charts showing the proportion of promoters vs distal sites in the specific sites for the FOS ChIP shown in Figure 5B. **(E)** Average profiles showing the tag count, scaled to average peak height, of PolII with siMM or siRUNX1::ETO[12] and ATAC-seq with or without doxycycline induction of shRUNX1::ETO at the FOS sites gained and lost with shRUNX1::ETO shown in Figure 5B. **(F)** ChIP for FOS and RUNX1::ETO in Kasumi-1 were ranked by fold change and represented as a density plot. RUNX1 ChIP, AP-1 and RUNX motifs were plotted on the same axis, +/-1kb of the summit. The purple bar indicates RUNX1::ETO specific sites and the blue bar FOS specific sites where the normalised tag-count is at least two-fold different. **(G)** ChIP for H3K9acS10P (note: antibody may also cross-react with H3K9acS28P) was performed with and without shRUNX1::ETO induced by doxycycline in the Kasumi-1 cell line, ranked by fold change of the tag count at promoters and represented as density plots (+/2kb of the summit). The red bar indicates shRUNX1::ETO specific sites and the green bar control specific sites where the normalised tag-count of specific sites is at least two-fold different. FOS ChIP (from Figure 5B) and H3K9ac +/- siRUNX1::ETO[12] are plotted along the same axis across the same window. **(H)** Sequences underlying the t(8;21) specific peaks at the *IL5RA* locus with the motif sequences highlighted. **(I)** Violin plots showing the expression per cell of *CSF2RB* in CEBPAx2 AML, grouped by LSCs and blasts[10]. **(J)** Normalised log_2 FPKM of *CSF2RB* in AML with different driver mutations and healthy CD34+ PBSCs[7]. Horizontal bars indicate the median of all samples. N = 4 CEBPAx2, 3 inv(16), 2 inv(3), 3 FLT3-ITD, 2 FLT3-ITD2x/NPM1, 3 NPM1, 2 PBSCs, 9 RUNX1 and 5 t(8;21) patients.
