# Supplementary material for: Leukemic stem cells activate lineage inappropriate signalling pathways to promote their growth
Source: Nat Commun. 2024 Feb 14;15:1359. doi: 10.1038/s41467-024-45691-4 (PMC10867020; doi:10.1038/s41467-024-45691-4)
Supplement: Supplementary file 2 — Reporting Summary [file 41467_2024_45691_MOESM2_ESM.pdf]

nature portfolio

Corresponding author(s): Sophie Kellaway, Constanze Bonifer

Last updated by author(s): Jan 4, 2024

Reporting Summary

Nature Portfolio wishes to improve the reproducibility of the work that we publish. This form provides structure for consistency and transparency in reporting. For further information on Nature Portfolio policies, see our Editorial Policies and the Editorial Policy Checklist.

Statistics

For all statistical analyses, confirm that the following items are present in the figure legend, table legend, main text, or Methods section.

n/a	Confirmed	
☐	☒	The exact sample size (n) for each experimental group/condition, given as a discrete number and unit of measurement
☐	☒	A statement on whether measurements were taken from distinct samples or whether the same sample was measured repeatedly
☐	☒	The statistical test(s) used AND whether they are one- or two-sided<br>*Only common tests should be described solely by name; describe more complex techniques in the Methods section.*
☐	☒	A description of all covariates tested
☐	☒	A description of any assumptions or corrections, such as tests of normality and adjustment for multiple comparisons
☐	☒	A full description of the statistical parameters including central tendency (e.g. means) or other basic estimates (e.g. regression coefficient) AND variation (e.g. standard deviation) or associated estimates of uncertainty (e.g. confidence intervals)
☐	☒	For null hypothesis testing, the test statistic (e.g. F, t, r) with confidence intervals, effect sizes, degrees of freedom and P value noted<br>*Give P values as exact values whenever suitable.*
☒	☐	For Bayesian analysis, information on the choice of priors and Markov chain Monte Carlo settings
☒	☐	For hierarchical and complex designs, identification of the appropriate level for tests and full reporting of outcomes
☒	☐	Estimates of effect sizes (e.g. Cohen's d, Pearson's r), indicating how they were calculated

Our web collection on statistics for biologists contains articles on many of the points above.

Software and code

Policy information about availability of computer code

Data collection	Flow cytometry collection software: BD FACSDiva, Summit 4.3, Attune NxT software
Data analysis	R versions 4.0.3, 4.1.0, 4.1.2. Trimmomatic 0.39. HISAT2.2.1. SAMtools 1.12. StringTie 2.1.3. Subread. Bowtie2 2.4.4. Picard 2.21.1. DeepTools 3.5.0. MACS2 2.2.7.1. BEDTools 2.29.2. HOMER 4.11. GSEA. EdgeR, LimmaVoom (Bioconductor). Seurat 4.1.0. Monocle3 1.0.0. flowCore 2.10.0. FlowJo v10

For manuscripts utilizing custom algorithms or software that are central to the research but not yet described in published literature, software must be made available to editors and reviewers. We strongly encourage code deposition in a community repository (e.g. GitHub). See the Nature Portfolio guidelines for submitting code & software for further information.

Data

Policy information about availability of data

All manuscripts must include a data availability statement. This statement should provide the following information, where applicable:

- Accession codes, unique identifiers, or web links for publicly available datasets
- A description of any restrictions on data availability
- For clinical datasets or third party data, please ensure that the statement adheres to our policy

RNA-seq, scRNA-seq, ATAC-seq, DNaseI-seq and ChIP seq data generated in this study have been deposited in the Gene Expression Omnibus (GEO) under accession code GSE226603 https://www.ncbi.nlm.nih.gov/geo/query/acc.cgi?&acc=GSE226603. Data from cell growth assays, gate percentages from flow cytometry and qPCR data are provided in the Source Data file. Published processed data was obtained from GSE1083167 https://www.ncbi.nlm.nih.gov/geo/query/acc.cgi?

acc=GSE108316 and the Human Cell Atlas https://explore.data.humancellatlas.org/projects/455b46e6-d8ea-4611-861e-de720a562ada. Published raw data was obtained from GSE1083167 https://www.ncbi.nlm.nih.gov/geo/query/acc.cgi?acc=GSE108316, GSE21109510 https://www.ncbi.nlm.nih.gov/geo/query/acc.cgi?acc=GSE211095 and GSE2922512 https://www.ncbi.nlm.nih.gov/geo/query/acc.cgi?acc=GSE29225. Human genome hg38 was downloaded from Ensembl https://www.ensembl.org/info/data/ftp/index.html

Research involving human participants, their data, or biological material

Policy information about studies with human participants or human data. See also policy information about sex, gender (identity/presentation), and sexual orientation and race, ethnicity and racism.

Reporting on sex and gender	No information on sex/gender was collected as samples were anonymised
Reporting on race, ethnicity, or other socially relevant groupings	No information on race, ethnicity or other socially relevant groupings was collected as samples were anonymised
Population characteristics	Diagnosis of AML with t(8;21) was the only characteristic required for this study and the only characteristic recorded
Recruitment	Participants were recruited based on diagnosis of AML with t(8;21) and capacity for informed consent
Ethics oversight	The study protocol was approved by the NHS National Research Ethics Committee

Note that full information on the approval of the study protocol must also be provided in the manuscript.

Field-specific reporting

Please select the one below that is the best fit for your research. If you are not sure, read the appropriate sections before making your selection.

[x] Life sciences [ ] Behavioural & social sciences [ ] Ecological, evolutionary & environmental sciences

For a reference copy of the document with all sections, see nature.com/documents/nr-reporting-summary-flat.pdf

Life sciences study design

All studies must disclose on these points even when the disclosure is negative.

Sample size	Sample sizes usually covered several thousand data-points (cell numbers for flow cytometry) or data-points from genome-wide assays and were therefore deemed sufficient, no sample size calculation was performed but coverage allows detection of changes even in a single site. Four independent primary samples and/or two cell lines and/or patient-derived xenograft were used for growth experiments, based on material availability. Sample size for in vivo experiments was based on technical limitations, power calculation suggests between 13 and 23 mice would be required for the effect size observed. For in vitro experiments no statistical method was used to predetermine the sample size and no data were excluded from the analyses.
Data exclusions	Mice which did not survive until the experimental end-point were not sampled for intermediate analysis. Data were excluded based on contaminating tumour cells for mice with leg tumours and 1 mouse with no human cells detected was excluded. No in vitro data was excluded from analysis.
Replication	All growth experiments were conducted at least three times, with mean, s.e.m. and Student's T tests/one way ANOVA/two way ANOVA used to determine if results were reproducible. Reproducibility of genome-wide data was assessed by correlating replicates, and by comparing specific features between independent biological samples. All replications were successful and included in final analysis.
Randomization	Mice were randomised for treatment with vehicle control or inhibitory antibodies. No other randomization was relevant for this type of study as patient data was combined and cell lines are a homogeneous population.
Blinding	Blinding during collection was not relevant for this type of study as outcomes are not apparent until after analysis using standardised pipelines of the raw data.

Reporting for specific materials, systems and methods

We require information from authors about some types of materials, experimental systems and methods used in many studies. Here, indicate whether each material, system or method listed is relevant to your study. If you are not sure if a list item applies to your research, read the appropriate section before selecting a response.

Materials & experimental systems

n/a	Involved in the study	
☐	☒	Antibodies
☐	☒	Eukaryotic cell lines
☒	☐	Palaeontology and archaeology
☐	☒	Animals and other organisms
☒	☐	Clinical data
☒	☐	Dual use research of concern
☒	☐	Plants

Methods

n/a	Involved in the study	
☐	☒	ChIP-seq
☐	☒	Flow cytometry
☐	☐	MRI-based neuroimaging

Antibodies

Antibodies used

Mouse anti-CD34-PE-Cy7 BD Biosciences Cat# 348811, RRID:AB_2868855
Mouse anti-human CD38-V450 BD Biosciences Cat# 646851, RRID:AB_1937282
Mouse anti-lineage cocktail-FITC BD Biosciences Cat# 340546, RRID:AB_400053
7-AAD staining solution BD Biosciences Cat# 559925, RRID:AB_2869266
Human anti-CD309 (VEGFR-2/KDR)-APC Miltenyi Biotec Cat# 130-117-984, RRID:AB_2733307
Human anti-CD125 (IL5RA)-biotin Miltenyi Biotec Cat# 130-110-543, RRID:AB_2654803
Streptavidin-PE-Cy7 eBioscience/Thermo Fisher Cat# 25-4317-82, RRID:AB_10116480
Mouse anti-FLAG Sigma-Aldrich/Merck Cat# F3165, RRID:AB_259529
Goat anti-mouse Alexa Fluor 594 Jackson ImmunoResearch Cat# 115-585-062, RRID:AB_2338876
Rabbit anti-FOS Invitrogen/Thermo Fisher Cat# MA5-15055, RRID:AB_10984728
Rabbit anti-CEBPA Santa Cruz Cat# sc-61X, RRID:AB_631233
Rabbit anti-RUNX1 Abcam Cat# ab23980, RRID:AB_2184205
Rabbit anti-RUNX1::ETO Diagenode Cat# C15310197, RRID:AB_2891230
Rabbit anti-PU.1 Santa Cruz Cat# sc-352, RRID:AB_632289
Goat anti-GATA2 R & D Systems Cat# AF2046, RRID:AB_355123
Rabbit anti-H3K27ac Abcam Cat# ab4729, RRID:AB_2118291
Rabbit anti-H3K9acS10P Abcam Cat# ab12181, RRID:AB_298913
Rabbit anti-H3K4me3 Millipore Cat# 04-745, RRID:AB_1163444
Rabbit anti-H3K27me3 Cell Signaling Technology Cat# 9733, RRID:AB_2616029
Mouse anti-human CD45-89Y Standard BioTools Cat# 3089003, RRID:AB_2661851
Mouse anti-human CD34-148Nd Standard BioTools Cat# 3148001B, RRID:AB_2810243
Mouse anti-human CD38-167Er Standard BioTools Cat# 3167001B, RRID:AB_2802110
Mouse anti-human Ki-67-172Yb Standard BioTools Cat# 3172024B, RRID: AB_2858243
Mouse anti-human CD117 BioLegend Cat# 313202, RRID: AB_314981
Rabbit anti-pSTAT1 (Y701)-153Eu Standard BioTools Cat# 3153003A, RRID: AB_2811248
Mouse anti-pSTAT3 (Y705)-158Gd Standard BioTools Cat# 3158005A, RRID: AB_2811100
Mouse anti-pSTAT5 (Y694)-150Nd Standard BioTools Cat# 3150005A, RRID: AB_2744690
Mouse anti-pS6 (S235/S236)-175Lu Standard BioTools Cat# 3175009A, RRID: AB_2811251
Rabbit anti-pCREB (S133)-176Yb Standard BioTools Cat# 3176005A, RRID:AB_2934290
Mouse anti-pNFκB-p65 (S529)-166Er Standard BioTools Cat# 3166006A, RRID: AB_2847867
Mouse anti-IκBα-164Dy Standard BioTools Cat# 3164004A, RRID: AB_2811249
Rabbit anti-p4E-BP1 (T37/T46)-149Sm Standard BioTools Cat# 3149005A, RRID: AB_2847866
Rabbit anti-p-Jnk1/Jnk2 (T183/Y185) ThermoFisher Cat# 700031, RRID: AB_2532273
Rabbit anti-p-cJun (S243) ThermoFisher Cat# PA5-104747, RRID: AB_2816220
Mouse anti-human beta2-microglobulin Biolegend Cat# 316302, RRID: AB_492835
Mouse anti-human CD298 Biolegend Cat# 341712, RRID: AB_2876646
Human anti-IL5RA-PE Miltenyi Biotec Cat# 130-110-602, RRID: AB_2654800
Human anti-hCD34-APC Miltenyi Biotec Cat# 130-120-519, RRID: AB_2811342
Human anti-hCD34-PE Miltenyi Biotec Cat# 130-120-515, RRID: AB_2811338
Rat anti-hCD11b-APC Miltenyi Biotec Cat# 130-091-241, RRID: AB_244268
Human anti-hCD309 (VEGFR-2)-APC-Vio770 Miltenyi Biotec Cat# 130-117-987, RRID: AB_2733085
Mouse anti-hCD45-APC-eFluor 780 ThermoFisher (eBio) Cat# 47-0459-42, RRID: AB_1944368
Mouse anti-CD33-BV421 Biolegend Cat# 303415
Bevacizumab Selleck Chemicals CAS 216974-75-3
Benralizumab AstraZeneca, gift N/A

Validation

Mouse anti-CD34-PE-Cy7, Mouse anti-human CD38-V450, Mouse anti-lineage cocktail-FITC Validatin by BD Biosciences: The specificity is confirmed by using multiple applications that may include a combination of flow cytometry, immunofluorescence, immunohistochemistry or western blot to test a combination of primary cells, cell lines or transfectant models. All flow cytometry reagents are titrated on the relevant positive or negative cells.

Human anti-CD309 (VEGFR-2/KDR)-APC Validation by Miltenyi Biotec: Human umbilical vein endothelial cells (HUVECs) were stained with CD309 (VEGFR-2) antibodies or with the correspondong REA Control (S) antibodies and analyzed by flow cytometry using the

MACSQuant® Analyzer. Extended validation was by epitope competition assay and comparison to other commercially available hybridoma clones.

Human anti-CD125 (IL5RA)-biotin Validation by Miltenyi Biotec: Human peripheral blood cells after erythrocyte lysis were stained with CD125 (IL-5Rα) antibodies or with the corresponding REA Control (S) antibodies as well as with Anti-Siglec-8 antibodies. CD11b+ cells were pre-gated for the analysis. Flow cytometry was performed using the MACSQuant® Analyzer. Extended validation was by epitope competition assay and comparison to other commercially available hybridoma clones.

Mouse anti-FLAG Validation by Sigma-Aldrich by dotblot and western blot

Goat anti-mouse Alexa Fluor 594 validation by Jackson: Based on immunoelectrophoresis and/or ELISA, the antibody reacts with whole molecule mouse IgG. It also reacts with the light chains of other mouse immunoglobulins. No antibody was detected against non-immunoglobulin serum proteins. The antibody has been tested by ELISA and/or solid-phase adsorbed to ensure minimal cross-reaction with human, bovine and horse serum proteins, but it may cross-react with immunoglobulins from other species.

Rabbit anti-FOS Validation by ThermoFisher: This Antibody was verified by Cell treatment to ensure that the antibody binds to the antigen stated. Western blot analysis of c-Fos using c-Fos Monoclonal Antibody (T.142.5) (Product # MA5-15055) shows increased expression of c-Fos in A-431 and NIH/3T3 cell lines upon EGF and Serum Starvation followed by Serum Release treatments.

Rabbit anti-RUNX1 Validation by Abcam: western blot. In house validation: Ptasinska et al. 2012, Ptasinska et al. 2014, Kellaway et al. 2021.

Rabbit anti-RUNX1::ETO Validation by Diagenode: Western blot, ELISA and ChIP in Kasumi-1

Goat anti-GATA2 Validation by R & D Systems: western blot, immunocytochemistry and immunohistochemistry in multiple haematopoietic cell lines

Rabbit anti-H3K27ac, Rabbit anti-H3K9acS10P Validation by Abcam: western blot and ChIP, note H3K9acS28P may cross reaction with S28P as determined with knockdown experiments by Abcam

Rabbit anti-H3K4me3 validated by Millipore by western blot, ChIP-seq. Routinely evaluated by immunoblot on in acid extracted proteins from HeLa cells, but not recombinant unmethylated Histone H3.

Rabbit anti-H3K27me3 validation by Cell Signaling Technology by western blot and SimpleChIP® Enzymatic Chromatin IP Kits. The CUT&RUN dilution was determined using CUT&RUN Assay Kit #86652.

Mouse anti-human CD45-89Y ,Mouse anti-human CD34-148Nd, Mouse anti-human CD38-167Er, Mouse anti-human Ki-67-172Yb, Rabbit anti-pSTAT1 (Y701)-153Eu, Mouse anti-pSTAT3 (Y705)-158Gd, Mouse anti-pSTAT5 (Y694)-150Nd, Mouse anti-pS6 (S235/S236)-175Lu, Rabbit anti-pCREB (S133)-176Yb, Mouse anti-pNFκB-p65 (S529)-166Er, Mouse anti-IκBα-164Dy , Rabbit anti-p4E-BP1 (T37/T46)-149Sm validation by Standard Biotools: Bagwell et al. Cytometry Part B Clinical Cytometry 98 (2020): 146–160.

Mouse anti-human CD117, mouse anti-human CD298, mouse anti-human beta2-microglobulin validation by Biolegend: Each lot of this antibody is quality control tested by immunofluorescent staining with flow cytometric analysis

Rabbit anti-p-Jnk1/Jnk2 (T183/Y185) Validation by ThermoFisher: western blot, ChIP, flow cytometry. Advanced verification by western blotting analysis of Phospho-JNK1/JNK2 (Thr183, Tyr185) using Anti-Phospho-JNK1/JNK2 (Thr183, Tyr185) Antibody (D12H7L17), Recombinant Rabbit Monoclonal (Product # 700031) shows increased expression of Phospho-JNK1/JNK2 (Thr183, Tyr185) in MCF7 cells treated with Anisomycin.

Rabbit anti-p-cJun (S243) Validation by ThermoFisher: Western blot analysis of Phospho-c-Jun (Ser243) in mouse brain/rat brain (absence (-) or presence (+) of non-phospho and phospho peptide).

Human anti-IL5RA-PE Validation by Miltenyi Biotec: Human peripheral blood cells after erythrocyte lysis were stained with CD125 (IL-5Rα) antibodies or with the corresponding REA Control (S) antibodies as well as with Anti-Siglec-8 antibodies. CD11b+ cells were pre-gated for the analysis. Flow cytometry was performed using the MACSQuant® Analyzer. Extended validation was by epitope competition assay and comparison to other commercially available hybridoma clones.

Human anti-hCD34-APC, hCD34-PE Validation by Miltenyi Biotec: Human peripheral blood cells (PBMCs) were stained with CD34 antibodies or with the corresponding REA Control (S) antibodies (left images) as well as with CD45 antibodies. CD45+ cells were pre-gated for the analysis. Flow cytometry was performed using the MACSQuant® Analyzer. Extended validation was by epitope competition assay and comparison to other commercially available hybridoma clones.

Rat anti-hCD11b-APC Validation by Miltenyi Biotec: Mouse spleenocytes were stained with CD11b antibodies or with the corresponding isotype control antibodies (left images) and analyzed by flow cytometry using the MACSQuant® Analyzer. Extended validation was by epitope competition assay and comparison to other commercially available hybridoma clones.

Human anti-hCD309 (VEGFR-2)-APC-Vio770 Validation by Miltenyi Biotec: Human umbilical vein endothelial cells (HUVECs) were stained with CD309 (VEGFR-2) antibodies or with the correspondong REA Control (S) antibodies and analyzed by flow cytometry using the MACSQuant® Analyzer. Extended validation was by epitope competition assay and comparison to other commercially available hybridoma clones.

Mouse anti-hCD45-APC-eFluor 780 Validation by ThermoFisher by flow cytometry, with advanced validation antibody clone (HI30)

specificity was demonstrated by CRISPR-Cas9 mediated knockout of target protein. Loss of signal was observed for target protein in HI30 KOcells compared to the control Cas9cells using CD45 antibody (HI30).

CD33-BV421 validation by Biolegend: Each lot of this antibody is quality control tested by immunofluorescent staining with flow cytometric analysis on human monocytes.

Bevacizumab and Benralizumab are FDA-approved monoclonal antibody therapeutics.

In addition all antibodies used for transcription factor ChIP (FOS, CEBPA, RUNX1, RUNX1::ETO, PU.1, GATA2) were confirmed to have enrichment of the consensus binding motif in de novo motif searches.

Eukaryotic cell lines

Policy information about cell lines and Sex and Gender in Research

Cell line source(s)	Kasumi-1, SKNO-1, HEK293T, MOLM14, MV411 and U937 were obtained from DMSZ.
Authentication	Kasumi-1 and SKNO-1 were confirmed to possess the t(8;21) translocation by gene expression analysis using fusion-specific primers. HEK293T, MOLM-14, MV4-11 and U937 were not authenticated.
Mycoplasma contamination	Cell lines were regularly tested negative for mycoplasma
Commonly misidentified lines (See ICLAC register)	No commonly misidentified cell lines were used in this study

Animals and other research organisms

Policy information about studies involving animals; ARRIVE guidelines recommended for reporting animal research, and Sex and Gender in Research

Laboratory animals	NSG mice (NOD.Cg-Prkdcscid Il2rg tm1Wjl/SzJ) aged between 12 and 16 weeks, newborn MISTRG mice.
Wild animals	The study did not involve wild animals
Reporting on sex	Male and female mice were used to generate PDX, for drug treatment experiments male mice only were used as (1) the PDX engrafts in the ovaries of females resulting in highly variable latency end points and often before significant bone marrow engraftment and, (2) the bone marrow volume of males is larger than females so more cells could be harvested for analysis.
Field-collected samples	The study did not involve samples collected from the field
Ethics oversight	All mouse studies were carried out in accordance with UK Animals (Scientific Procedures) Act, 1986 under project licence P74687DB5 following approval from Newcastle University animal ethical review body (AWERB)

Note that full information on the approval of the study protocol must also be provided in the manuscript.

Plants

Seed stocks	*Report on the source of all seed stocks or other plant material used. If applicable, state the seed stock centre and catalogue number. If plant specimens were collected from the field, describe the collection location, date and sampling procedures.*
Novel plant genotypes	*Describe the methods by which all novel plant genotypes were produced. This includes those generated by transgenic approaches, gene editing, chemical/radiation-based mutagenesis and hybridization. For transgenic lines, describe the transformation method, the number of independent lines analyzed and the generation upon which experiments were performed. For gene-edited lines, describe the editor used, the endogenous sequence targeted for editing, the targeting guide RNA sequence (if applicable) and how the editor was applied.*
Authentication	*Describe any authentication procedures for each seed stock used or novel genotype generated. Describe any experiments used to assess the effect of a mutation and, where applicable, how potential secondary effects (e.g. second site T-DNA insertions, mosiacism, off-target gene editing) were examined.*

ChIP-seq

Data deposition

- [x] Confirm that both raw and final processed data have been deposited in a public database such as GEO.
- [x] Confirm that you have deposited or provided access to graph files (e.g. BED files) for the called peaks.

Data access links *May remain private before publication.*	ChIP seq data produced in this study have been deposited in the Gene Expression Omnibus (GEO) under accession code GSE226531 and can be accessed using the reviewer access token wnuliaiozxshnml

Files in database submission	CEBPA_CTRL.bdg, CEBPA_dnFOS.bdg, ETO_CTRL.bdg, ETO_dnFOS.bdg, FOS_dnFOS_CTRL.bdg, FOS_dnFOS_plusdnFOS.bdg, FOS_minusBEV.bdg, FOS_plusBEV.bdg, FOS_shRE_CTRL.bdg, FOS_shRE_shRE.bdg, GATA2_CTRL.bdg, GATA2_dnFOS.bdg, H3K27ac_CTRL.bdg, H3K27ac_dnFOS.bdg, H3K27me3_CTRL.bdg, H3K27me3_dnFOS.bdg, H3K4me3_CTRL.bdg, H3K4me3_dnFOS.bdg, H3K9acS10P_CTRL.bdg, H3K9acS10P_shRE.bdg, PU1_CTRL.bdg, PU1_dnFOS.bdg, RUNX1_CTRL.bdg, RUNX1_dnFOS.bdg
Genome browser session (e.g. UCSC)	http://genome-euro.ucsc.edu/cgi-bin/hgTracks?hubUrl=https://genome-trackhub.bham.ac.uk/data/t821signalling/hub.txt&genome=hg38&position=lastDbPos

Methodology

Replicates

ChIP-seq experiments were conducted on 2-20 million cells, with 2-4 individual immunoprecipitations pooled to generate the sequencing libraries. Replicates of sequencing libraries were not conducted.

Sequencing depth

All ChIP-seq experiments were sequenced single-end with 75bp reads. Number of reads indicated for each sample, given as total/uniquely mapped.
CEBPA_CTRL: 34275518/28783984
CEBPA_dnFOS: 30678827/25863115
ETO_CTRL: 31216374/22042706
ETO_dnFOS: 32629391/10189199
FOS_dnFOS_CTRL: 34511100/24133914
FOS_dnFOS_plusdnFOS: 25754517/8744590
FOS_minusBEV: 31248365/11264323
FOS_plusBEV: 32264215/14368937
FOS_shRE_CTRL: 23164582/19794542
FOS_shRE_shRE: 19994914/14445566
GATA2_CTRL: 30847642/6575157
GATA2_dnFOS: 49809323/11999106
H3K27ac_CTRL: 60119196/35105088
H3K27ac_dnFOS: 64644672/40341437
H3K27me3_CTRL: 9146316/8923212
H3K27me3_dnFOS: 8710757/8519565
H3K4me3_CTRL: 34091700/15321739
H3K4me3_dnFOS: 31215059/21829759
H3K9acS10P_CTRL: 30552070/28128740
H3K9acS10P_shRE: 24257682/20655158
PU1_CTRL: 62382474/34623722
PU1_dnFOS: 58220191/46384959
RUNX1_CTRL: 34618569/22860662
RUNX1_dnFOS: 11881388/5091501

Antibodies

Rabbit anti-FOS Invitrogen/Thermo Fisher Cat# MA5-15055, RRID:AB_10984728
Rabbit anti-CEBPA Santa Cruz Cat# sc-61X, RRID:AB_631233
Rabbit anti-RUNX1 Abcam Cat# ab23980, RRID:AB_2184205
Rabbit anti-RUNX1::ETO Diagenode Cat# C15310197, RRID:AB_2891230
Rabbit anti-PU.1 Santa Cruz Cat# sc-352, RRID:AB_632289
Goat anti-GATA2 R & D Systems Cat# AF2046, RRID:AB_355123
Rabbit anti-H3K27ac Abcam Cat# ab4729, RRID:AB_2118291
Rabbit anti-H3K9acS10P Abcam Cat# ab12181, RRID:AB_298913
Rabbit anti-H3K4me3 Millipore Cat# 04-745, RRID:AB_1163444
Rabbit anti-H3K27me3 Cell Signaling Technology Cat# 9733, RRID:AB_2616029

Peak calling parameters

Peaks were called using MACS2 using the settings -q 0.01 -B --trackline

Data quality

Default mfold parameter of 10 was used, and q-value cut-off of 0.01

Software

ChIP-sequencing reads were processed with Trimmomatic v0.39 to remove sequencing adaptors and low quality reads. Trimmed reads were aligned to the human genome (version hg38) using Bowtie2 v2.3.5.1 using the setting --very-sensitive-local. PCR duplicates were removed using the MarkDuplicates function Picard v2.21.1. Density plots were generated using Homer v4.11 annotatePeaks.pl function using the bedGraph files generated by MACS2, with the options -size 2000 -hist 10 -ghist and plotted using JavaTreeView 1.1.6.

Flow Cytometry

Plots

Confirm that:

- [x] The axis labels state the marker and fluorochrome used (e.g. CD4-FITC).
- [x] The axis scales are clearly visible. Include numbers along axes only for bottom left plot of group (a 'group' is an analysis of identical markers).
- [x] All plots are contour plots with outliers or pseudocolor plots.
- [x] A numerical value for number of cells or percentage (with statistics) is provided.

Methodology

Sample preparation	Flow cytometry was carried out on cell lines, prepared by aspirating cells from culture and washing in PBS/BSA/EDTA, primary AML samples taken from bone marrow (all sequencing experiments) or blood (proliferation assay) which underwent lymphoprep to isolate mononuclear cells followed by washing in PBS/BSA, and on patient-derived xenograft cells harvested from mouse bone marrow by maceration, followed by washing in PBS/BSA/EDTA.
Instrument	Cell lines were analysed on a Cyan ADP, patient-derived xenograft on an Attune NxT, and primary AML samples were sorted on a FACS Aria
Software	Data collection used BD FACSDiva, Summit 4.3, Attune NxT software, analysis was carried out using FlowJo v10.
Cell population abundance	t(8;21)#1: CD34+CD38+ 1,630,000 (58.5%), CD34+CD38- 753,000 (32%)<br>t(8;21)#2: Sort 1 (scRNA-seq, CFU-assay) CD34+CD38+ 701,181 (90.4%), CD34+CD38- 37,732 (6%), sort 2 (bulk RNA-seq, ATAC-seq) CD34+CD38+ 810,584, CD34+CD38- 40,412, sort 3 (blood for proliferation assay) CD34+CD38+ 598,817, CD34 +CD38- 261,664. Precision values of 16-32-0 were used to ensure purity.
Gating strategy	Debris was removed by gating the dominant cell population on FSC/SSC and doublets were removed by gating the main cell population on FSC-A/FSC-H. To sort LSCs and Blasts, live cells were gated on 7-AAD with a positive and negative control. Lineage positive cells were also gated out based on lineage cocktail FITC, then CD34+ cells were selected and from this population, CD38+/- with the gates for these markers set based on isotype controls, sorted populations shown in Extended Data Figure 1. For analysis of patient-derived xenograft cells from bone marrow, debris and doublets were gated as above, then hCD45+ cells selected. Gating for CD34+CD38+/- cells followed as above based on isotype controls, and KDR/IL5RA cells visualised from these gates, shown in Figure 4.

- [x] Tick this box to confirm that a figure exemplifying the gating strategy is provided in the Supplementary Information.

Magnetic resonance imaging

Experimental design

Design type	*Indicate task or resting state; event-related or block design.*
Design specifications	*Specify the number of blocks, trials or experimental units per session and/or subject, and specify the length of each trial or block (if trials are blocked) and interval between trials.*
Behavioral performance measures	*State number and/or type of variables recorded (e.g. correct button press, response time) and what statistics were used to establish that the subjects were performing the task as expected (e.g. mean, range, and/or standard deviation across subjects).*

Acquisition

Imaging type(s)	*Specify: functional, structural, diffusion, perfusion.*
Field strength	*Specify in Tesla*
Sequence & imaging parameters	*Specify the pulse sequence type (gradient echo, spin echo, etc.), imaging type (EPI, spiral, etc.), field of view, matrix size, slice thickness, orientation and TE/TR/flip angle.*
Area of acquisition	*State whether a whole brain scan was used OR define the area of acquisition, describing how the region was determined.*

Diffusion MRI ☐ Used ☐ Not used

Preprocessing

Preprocessing software	*Provide detail on software version and revision number and on specific parameters (model/functions, brain extraction, segmentation, smoothing kernel size, etc.).*

Normalization | *If data were normalized/standardized, describe the approach(es): specify linear or non-linear and define image types used for transformation OR indicate that data were not normalized and explain rationale for lack of normalization.*

Normalization template | *Describe the template used for normalization/transformation, specifying subject space or group standardized space (e.g. original Talairach, MNI305, ICBM152) OR indicate that the data were not normalized.*

Noise and artifact removal | *Describe your procedure(s) for artifact and structured noise removal, specifying motion parameters, tissue signals and physiological signals (heart rate, respiration).*

Volume censoring | *Define your software and/or method and criteria for volume censoring, and state the extent of such censoring.*

Statistical modeling & inference

Model type and settings | *Specify type (mass univariate, multivariate, RSA, predictive, etc.) and describe essential details of the model at the first and second levels (e.g. fixed, random or mixed effects; drift or auto-correlation).*

Effect(s) tested | *Define precise effect in terms of the task or stimulus conditions instead of psychological concepts and indicate whether ANOVA or factorial designs were used.*

Specify type of analysis: ☐ Whole brain ☐ ROI-based ☐ Both

Statistic type for inference | *Specify voxel-wise or cluster-wise and report all relevant parameters for cluster-wise methods.*

(See Eklund et al. 2016)

Correction | *Describe the type of correction and how it is obtained for multiple comparisons (e.g. FWE, FDR, permutation or Monte Carlo).*

Models & analysis

n/a	Involved in the study
☐	☐ Functional and/or effective connectivity
☐	☐ Graph analysis
☐	☐ Multivariate modeling or predictive analysis

Functional and/or effective connectivity | *Report the measures of dependence used and the model details (e.g. Pearson correlation, partial correlation, mutual information).*

Graph analysis | *Report the dependent variable and connectivity measure, specifying weighted graph or binarized graph, subject- or group-level, and the global and/or node summaries used (e.g. clustering coefficient, efficiency, etc.).*

Multivariate modeling and predictive analysis | *Specify independent variables, features extraction and dimension reduction, model, training and evaluation metrics.*
