# Supplementary material for: Leukemic stem cells activate lineage inappropriate signalling pathways to promote their growth
Source: Nat Commun. 2024 Feb 14;15:1359. doi: 10.1038/s41467-024-45691-4 (PMC10867020; doi:10.1038/s41467-024-45691-4)
Supplement: Supplementary file 3 — Description of Additional Supplementary Files [file 41467_2024_45691_MOESM3_ESM.docx]

**Description of Additional Supplementary Files**

File Name: Supplementary Data 1

Description: LSC and blast specific genes (log2 0.5-fold, adjusted p <0.1) from primary AML patient scRNA-seq integrated dataset

File Name: Supplementary Data 2

Description: G1-stage LSC and Blast marker genes

File Name: Supplementary Data 3

Description: RNA fold changes following expression of dnFOS in Kasumi-1, log2 fold changes and adjusted p-values

File Name: Supplementary Data 4

Description: RNA fold changes following expression of dnFOS in PDX or healthy CD34+ cells
